# Serum Lipids, Inflammation, and the Risk of Atrial Fibrillation: Pathophysiological Links and Clinical Evidence

**DOI:** 10.3390/jcm14051652

**Published:** 2025-02-28

**Authors:** Alfredo Mauriello, Adriana Correra, Anna Chiara Maratea, Alfredo Caturano, Biagio Liccardo, Marco Alfonso Perrone, Antonio Giordano, Gerardo Nigro, Antonello D’Andrea, Vincenzo Russo

**Affiliations:** 1 Cardiology Unit, Department of Medical and Translational Sciences, University of Campania “Luigi Vanvitelli”, Monaldi Hospital, 80131 Naples, Italy; alfredo.mauriello93@libero.it (A.M.); annachiara.maratea@gmail.com (A.C.M.); liccardob@gmail.com (B.L.); gerardo.nigro@unicampania.it (G.N.); 2 Cardiology and Intensive Care Unit, Department of Cardiology, “Umberto I” Hospital, 84014 Nocera Inferiore, Italy; antonellodandrea@libero.it; 3 Intensive Cardiac Care Unit, “San Giuseppe Moscati” Hospital, ASL Caserta 81031 Aversa, Italy; adrianacorrera@gmail.com; 4 Internal Medicine Unit, Department of Advanced Medical and Surgical Sciences, University of Campania Luigi Vanvitelli, Piazza Luigi Miraglia 2, 80138 Naples, Italy; alfredo.caturano@uniroma5.it; 5 Department of Cardiology and CardioLab, University of Rome Tor Vergata, 00133 Rome, Italy; marco.perrone@uniroma2.it; 6 Sbarro Institute for Cancer Research and Molecular Medicine, Center for Biotechnology, College of Science and Technology, Temple University, Philadelphia, PA 19122, USA; antonio.giordano@temple.edu

**Keywords:** atrial fibrillation, dyslipidemia, statins, evolocumab, alirocumab, inclisiran, bempedoic acid, icosapent ethyl

## Abstract

Dyslipidemia is a metabolic disorder characterized by quantitative and/or qualitative abnormalities in serum lipid levels. Elevated serum cholesterol levels can modify the turnover and recruitment of ionic channels in myocytes and cellular homeostasis, including those of inflammatory cells. Experimental and clinical data indicate that inflammation is implicated in the pathophysiology of atrial remodeling, which is the substrate of atrial fibrillation (AF). Data about the association between increased lipid serum levels and AF are few and contrasting. Lipoprotein (a), adiposity, and inflammation seem to be the main drivers of AF; in contrast, low-density lipoproteins, high-density lipoproteins and triglycerides are not directly involved in AF onset. The present review aimed to describe the pathophysiological link between dyslipidemia and AF, the efficacy of lipid-lowering therapies in atherosclerotic cardiovascular disease (ASCVD) patients with and without AF, and the impact of lipid-lowering therapies on AF incidence.

## 1. Introduction

Dyslipidemia is a metabolic disorder characterized by quantitative and/or qualitative abnormalities in serum lipids levels. Quantitatively, dyslipidemia is due to elevated plasma total cholesterol (TC), low-density lipoprotein cholesterol (LDL-c), triglycerides (TG), and reduced high-density lipoprotein cholesterol (HDL-c) levels, either singly or in combination. Qualitatively, dyslipidemia implies changes in the composition of LDL-c, which includes small dense LDL-c, increased TG content, or increased electronegativity of LDL-c [1].

The prevalence of dyslipidemia varies widely due to differences in definitions across clinical studies and the demographic and clinical characteristics of studied populations, such as sex, age, ethnicity, and comorbidities [2]. While polygenic dyslipidemia is very common, affecting approximately 1 in 100 individuals, rare forms like Tangier disease or analphalipoproteinaemia are observed in fewer than 1 in 1,000,000 people [1]. Dyslipidemia is particularly prevalent in cardiovascular diseases, affecting up to 40% of individuals with established cardiovascular conditions and up to 80% of those experiencing acute coronary syndrome [3].

The association between the absolute changes in plasma LDL-c and TG levels and the risk of atherosclerotic cardiovascular disease (ASCVD) is well known [2]. Elevated serum LDL-c levels directly increase the risk of cardiovascular death, myocardial infarction, unstable angina, coronary revascularization, and stroke [4,5,6,7,8,9]. Additionally, elevated TG levels are an additional cause of cardiovascular disease and all-cause mortality [10]. However, the relationship between dyslipidemia and atrial fibrillation (AF), a major cardiovascular condition, remains unclear. While some evidence suggests a potential association between abnormal serum lipid levels and AF, data are sparse and often conflicting [11]. This review aims to elucidate the pathophysiological mechanisms linking dyslipidemia and AF, evaluate the efficacy of lipid-lowering therapies in ASCVD patients with and without AF, and explore their impact on AF incidence.

## 2. Atrial Fibrillation Pathogenesis

The pathogenesis of AF is complex, involving multiple interacting factors, including substrates, triggers, and modulators, which play a pivotal role in its development and persistence. Atrial remodeling (electrical, structural, and contractile) is central to most acquired forms of AF [12].

### 2.1. Electrical Remodeling

In a healthy atrium, the atrial cell action potentials (APs) remain at the resting potential after repolarization due to the activity of the inward rectifier potassium current (I_K1_), which overwhelms the pacemaker current (I_f_), preventing the manifest automaticity. From an electrophysiological perspective, AF onset is due to enhanced automaticity caused by changes in this balance resulting from decreased I_K1_ and/or enhanced I_f_ [13]. Moreover, early (EADs) and delayed (DADs) afterdepolarizations may enhance the cells automaticity. The main factor causing EADs is the prolonged AP duration, which allows L-type Ca^2+^ current (I_CaL_) to recover from inactivation, leading to depolarizing inward movement of Ca^2+^ ions [14]. In contrast, DADs are caused by abnormal diastolic release of Ca^2+^ from sarcoplasmic reticulum Ca^2+^ stores [15].

Reentrant circuits further sustain AF by forming rapidly firing drivers with fibrillatory propagation or multiple irregular pathways. Abnormalities in refractoriness and conduction velocity are the significant determinants of the substrates that enable AF reentry [16]. The refractory period (RP) is determined by the action potential duration, which is governed by the balance between inward (Ca^2+^ and Na^+^) and outward (K^+^) currents. Conduction velocity is determined by inward currents that provide depolarization energy (mainly Na^+^) and gap junction channels (connexins) that provide electrical continuity between cells [16]. Increased outward K^+^ currents, through K^+^ voltage-gated (Kv) channels, or decreased inward Ca^2+^ currents can reduce the RP, accelerating repolarization and promoting AF [16].

### 2.2. Structural Remodeling

Structural remodeling is mainly attributed to atrial fibrosis [17]. Interstitial fibrosis separates muscle bundles, while replacement fibrosis disrupts electrical continuity by replacing dead myocytes [18]. These disruptions slow conduction, facilitating reentry. Additionally, fibroblasts can electrically couple with cardiomyocytes, promoting abnormal electrical activity and reentrant circuits [18]. AF itself perpetuates structural remodeling by creating a long-term self-reinforcing cycle that contributes to developing persistent forms that sustain the arrhythmia [19].

### 2.3. Contractile Remodeling

Contractile atrial remodeling results from the close interplay between ventricular and atrial function. Ventricular dysfunction determines an increase in end-diastolic pressure with an increase in filling pressures; moreover, it determines a neurohormonal dysfunction [20]. The increase in filling pressures is responsible for atrial stretch, fibrosis, altered Ca^2+^ handling, and ionic current remodeling, which leads to AF. Conversely, AF causes the loss of atrial kick, a reduction in coronary flow, and an excessively rapid ventricular rate, which contribute to the atrial changes responsible for AF.

### 2.4. Role of the Autonomic Nervous System

The autonomic nervous system (ANS) is not only associated with cardiac rhythm regulation but also exerts an important role in both triggering and maintaining AF [21]. Increased vagal nerve activity enhances the acetylcholine-dependent potassium current (IKACh), shortening the action potential duration and stabilizing reentrant rotors [16,22]. Meanwhile, heightened beta-adrenergic receptor activation leads to increase diastolic calcium leak and promotes abnormal electrical activity originating from DADs by hyper-phosphorylating the ryanodine receptor 2 (RyR2) [16]. Patients with persistent AF exhibit increased atrial sympathetic nerve density, underscoring the role of ANS dysregulation in AF pathogenesis [22].

## 3. Serum Lipids and Inflammation

### 3.1. Cholesterol

Cholesterol is a major component of plasma membranes [23]; it regulates the surface expression of several ion channels [24] and intracellular homeostasis [25]. Elevated serum cholesterol levels can alter the turnover and recruitment of Kv channels in myocytes and cellular homeostasis, including those in inflammatory cells [26]. The relationship between high serum cholesterol levels and inflammation has been studied in cellular [27,28,29], animal [30,31,32,33], and human models [29,30,34].

In myeloid cells, cholesterol accumulation activates the NOD-like receptors (NLR) family pyrin domain containing 3 (NLRP3) inflammasome, which enhances neutrophil accumulation and neutrophil extracellular trap formation in atherosclerotic plaques [27]. Similarly, macrophages deficient in transporter adenosine triphosphate (ATP)-binding cassette subfamily A member 1 (ABCA1) and ATP-binding cassette subfamily G member 1 (ABCG1) exhibit intracellular cholesterol buildup, amplifying inflammatory gene responses to toll-like receptor 2, toll-like receptor 3, and toll-like receptor 4 ligands [28].

The relationship between dyslipidemia and innate immunity has been extensively studied in animal models [30,31,32].

In low-density lipoprotein receptor (LDL-r) knockout mice, haploinsufficiency of apolipoprotein A-1 (ApoA-1) reduces HDL levels, driving hematopoietic stem and progenitor cell (HSPC) expansion and monocyte proliferation in the bone marrow [31]. Furthermore, cholesterol accumulation in mice with efficient deletion of ABCA1 and ABCG1 leads to monocytes and neutrophils hyperproliferation, accompanied by heightened inflammatory gene expression [32].

Human studies support these findings. HSPCs from patients with elevated LDL-c levels display migratory gene profiles favoring bone marrow exit and a transcriptional bias towards monocyte proliferation, reversible with twelve-week cholesterol-lowering therapy [29]. Patients with ABCA1 mutations and low HDL-c levels exhibit increased serum levels of tumor necrosis factor-alpha (TNF-a) and interleukin (IL)-6, which seem to be attenuated by administration of statins [34].

In addition to inflammatory-induced electrical and anatomical changes, high levels of cholesterol have shown a direct role in the function of potassium [24] and calcium channels [33], contributing to arrhythmic risk. Cholesterol interacts with the channel protein, changing the physical properties of the membrane bilayer and maintaining the scaffolds for protein–protein interactions [24]. For example, LDL receptor knockout mice with elevated LDL-c levels exhibit increased L-type calcium currents, heightening arrhythmic susceptibility [33].

### 3.2. Triglycerides

Epidemiological data revealed a correlation between increased serum TG levels, C-reactive protein (C-RP), and cardiovascular risk [35,36].

Among 73,513 individuals from the Copenhagen Registry, each 1 mmol/L (39 mg/dL) increase in non-fasting remnant cholesterol particles (rich in TG) was associated with a 37% rise in C-RP levels [37] and a 2.8-fold increased risk for ischemic heart disease, independent of HDL-c levels [37].

Similarly, patients with familial dysbetalipoproteinemia with high levels of remnant cholesterol particles, rich in TG, show heightened monocyte-driven inflammation and arterial wall inflammation [36].

### 3.3. Lipoprotein (a)

Lp(a) is a lipoprotein involved in lipid transport, formed by the covalent linkage of apolipoproteins A and B through disulfide bonds [38]. Circulating levels of Lp(a) are genetically determined, with Lp(a) locus explaining most of the variance, up to 90% [39]. Lp(A) has a direct role in the inflammation of the vessels. In cultured human endothelial cells, there is an observed increase in vascular cell adhesion molecule 1 (VCAM-1) and E-selectin [40] and intercellular adhesion molecule 1 (ICAM-1) [41,42]. Furthermore, Lp(a) facilitates monocyte attachment and infiltration [43]. Also, Lp(a) accelerates chemotaxis indirectly by driving human endothelial cells to secrete monocyte chemotactic protein (MCP) [44].

This lipid-induced inflammatory state provides a plausible pathophysiological link between dyslipidemia and AF since it may modify the typical organization and function of atrial tissue, promoting the development of atrial fibrosis and electrical instability [45,46,47].

## 4. Epicardial Fat and Atrial Fibrillation

Epicardial adipose tissue (EAT) is the visceral fat depot of the heart located between the myocardium and the visceral layer of the pericardium [48,49]. EAT extends from the epicardial surface into the myocardium, often following the adventitia of the coronary artery branches without any fascial structures separating it from myocardial tissue [50]. High serum LDL-c and low HDL-c levels are strongly associated with increased EAT [51,52,53].

Several epidemiological studies showed a causal association between EAT and cardiovascular disease [54,55]. In particular, the doubling of EAT thickness was associated with a 1.5-fold risk of coronary events when adjusting for cardiovascular risk factors [56]. Moreover, EAT thickness has been associated with an increased risk of AF [57]. A meta-analysis of 63 observational studies, including 352,275 individuals, reported that a one SD increase in EAT volume was associated with a 2.6-fold risk of AF, a 2.1-fold risk of paroxysmal AF, and a 5.4-fold risk of persistent AF compared to sinus rhythm. Similarly, a one-SD higher EAT volume was linked to 2.2-fold higher odds of persistent versus paroxysmal AF and increased risks of post-ablation, post-operative, and post-cardioversion AF.

The causal relationship between EAT and AF involves several pathophysiological mechanisms [48].

EAT produces and secretes many pro-inflammatory cytokines (TNF-a, IL-6) and mediators (activin A and matrix metalloproteinases), which lead to pro-inflammatory and pro-fibrotic activity in the atria myocardium [48]. Inflammation has been proposed as one of the main pathogenetic mechanisms linking EAT and AF [53]. EAT shows an increased production of reactive oxygen species (ROS), leading to enhanced oxidative stress in adjacent myocardial tissue, possibly with the involvement of paracrine interactions [58]. The production of aromatase, which converts androgens into estrogen, appears to play an important role in modulating electromechanical properties [59].

In addition to these mechanisms, EAT could modify susceptibility to atrial arrhythmias by promoting ANS dysfunction. EAT is rich in autonomic ganglia and intercommunicating nerves, supported by high nerve growth factor production, which may act as a scaffold for cardiac autonomic nerves and ganglionated plexi [60]. The autonomic sympathetic ganglia within EAT can initiate rapid electrical discharging from the pulmonary veins or pulmonary veins–atrial junction, thus contributing to AF onset and maintenance [61]. It has been observed that the ablation of these structures may result in reducing AF recurrence [61]. Moreover, increased EAT thickness has been associated with elevated vagal tone [62].

**Figure 1:**
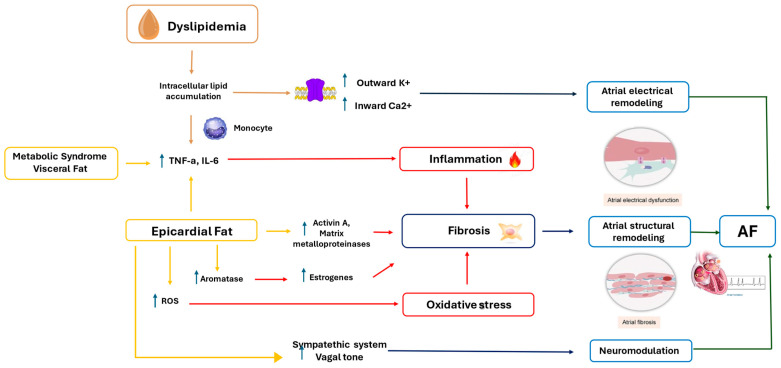
Mechanistic pathways linking dyslipidemia, epicardial fat, and atrial fibrillation. Dyslipidemia leads to intracellular lipid accumulation and inflammation through elevated TNF-α and IL-6 levels, promoting atrial electrical remodeling. Epicardial fat contributes to oxidative stress, increased ROS, and fibrosis via activin A, matrix metalloproteinases, and aromatase-mediated estrogen production. Fibrosis and oxidative stress result in atrial structural remodeling, neuromodulation, and eventual atrial fibrillation. AF: atrial fibrillation; IL-6: interleukin-6; ROS: reactive oxygen species; TNF-a: tumor necrosis factor-alpha.

The dysregulated production and secretion of adipokines by adipose tissue, particularly by visceral fat depots, may stimulate central sympathetic nervous system (SNS) activity, further promoting arrhythmic susceptibility [63]. The interplay between dyslipidemia, EAT, visceral fat, and AF highlights the multifactorial role of EAT in arrhythmogenesis. EAT promotes atrial fibrosis, electrical instability, and susceptibility to AF through its pro-inflammatory, oxidative, and neuro-modulatory effects. Figure 1 summarizes the linkage between dyslipidemia, EAT, visceral fat, and AF.

## 5. Visceral Adiposity, Metabolic Syndrome, and Atrial Fibrillation

Visceral adiposity (VAT) is an accumulation of fat surrounding the intra-abdominal or omental region [64]. Adipocytes constitute the main cellular component of adipose tissue and VAT contains a greater number of large adipocytes [65]. Large adipocytes are insulin resistant, hyperlipolytic, and resistant to the anti-lipolytic effect of insulin [66]. Lipids increase intake of visceral fat cells, determining a failure to maintain metabolic homeostasis of lipid storage versus lipolysis because lipid overload leads to endoplasmic reticulum stress, increased expression of inflammation regulator nuclear factor-kappa B (NF-kB), and the production of inflammation-inducing signals such as IL-6 and TNF-a [67,68]. VAT is associated with a high abundance of infiltrating macrophages and a proinflammatory secretome [64].

Metabolic syndrome (MetS) is a cluster of characteristics, including insulin resistance, central obesity, hypertension, and dyslipidemia, which themselves increase the risk of cardiovascular disease and adverse outcomes [69]. Visceral obesity is the most frequently observed component of MetS [70]. MetS has been associated with increased serum levels of IL-6 [71,72,73], TNF-α, CRP [72,73], and serum amyloid A (SAA) [73]. Weight loss leading to body mass index reduction was associated with a significant decrease in inflammatory biomarkers [71,72,73].

Among the general population, all five components of MetS (glucose intolerance, low levels of HDL-c, high levels of TG, obesity, and arterial hypertension), when diagnosed repeatedly, were independently associated with an increased risk of AF [74].

An observational study, including a total of 7,565,531 adults, with a mean follow-up of 7.9 years, showed a 31% increased risk of AF in patients with persistent MetS, a 26% increased risk in those with MetS who were healthy at the second evaluation, and a 16% increased risk in healthy patients newly diagnosed with MetS at the second evaluation compared with persistently healthy individuals [75].

## 6. Paradoxical Epidemiologic Association Between Serum Lipids and Atrial Fibrillation

Despite several pre-clinical data supporting the role of serum lipids in promoting inflammation and increasing AF risk [27,28,29], population-based clinical studies showed a paradoxical inverse association between serum lipid levels and AF risk [76,77,78].

Lp(a) seems to be the only lipid biomarker with robust causal evidence for the risk of AF [79,80].

In a community-based Japanese study, including 246,246 patients more than 40 years of age, hypercholesterolemia, defined as TC > 220 mg/dL or the use of cholesterol-lowering medication, was inversely associated with AF onset [77].

Similarly, a prospective study [76] of 88,785 Chinese patients, with an age range of 18 to 98 years, during a follow-up period of 7.12 years, demonstrated that higher TC and LDL-C levels were inversely associated with incident AF [76].

In a large population-based study, including 65,136 adults with a mean age of 42.6 years, from the Swedish National Patient Register and Cause of Death Register, higher levels of TC and LDL-c were associated with a reduced AF risk of 39% and 36% within the first 5 years, even if the effect was attenuated after 5 years of follow-up [78].

## 7. Drugs for the Treatment of Dyslipidemia and Their Role in Atrial Fibrillation

### 7.1. Statins

Statins reduce cholesterol synthesis in the liver by inhibiting the enzyme 3-hydroxy-3-methyl-glutaryl-coenzyme A (HMG-CoA) reductase. The reduction in intracellular cholesterol promotes increased LDL-r expression at the surface of the hepatocytes, resulting in increased cellular LDL uptake and decreased plasma concentrations of LDL and other apolipoprotein B (ApoB)-containing lipoproteins, including TG-rich particles [81].

The lipid-lowering effect of statins has a profound impact on cardiovascular outcomes. Specifically, for every 1 mmol/L reduction in LDL-C, statins reduce the risk of major vascular events—including myocardial infarction, death from coronary artery disease, stroke, or coronary revascularization—by 22%. They also lower the incidence of major coronary events by 23%, coronary artery disease-related mortality by 20%, total stroke by 17%, and overall mortality by 10% over a 5-year period [82].

Beyond their lipid-lowering effects, statins exhibit several pleiotropic properties [83], which may explain their ability to influence clinical outcomes independently of LDL-c reduction [83].

Several experimental studies have demonstrated that statins benefit various cell types. In endothelial cells, statins improve vasodilatory function through the production of nitric oxide [84]. In smooth muscle cells, statins help stabilize lipid plaque [85], while in inflammatory cells, they reduce the production of reactive oxygen species and inflammatory cytokines [86]. Additionally, statins reduce platelet aggregability and prevent excessive platelet activation. Finally, in cardiomyocytes, statins mitigate angiotensin-mediated fibrosis [87]. However, the relationship between statins and AF remains controversial and poorly understood, with limited and conflicting data available [83].

In a comprehensive review, involving 35 studies and a total of 1,277,306 patients with indication for lipid-lowering therapies, Oraii et al. revealed that statins reduced AF risk in patients with dyslipidemia and postoperative AF but showed no significant effect in preventing post-cardioversion AF. Moreover, they assessed the role of statins in both primary prevention (1,272,617 patients from 13 studies) and secondary prevention (4121 patients from 16 studies) of AF [88].

Regarding primary prevention, a meta-analysis considered six studies covering 766,556 patients with an AF risk. The use of statins was associated with a reduction in AF risk of between 19 and 57% [88]. Sixteen studies considered the efficacy of statins in preventing postoperative AF in 4121 patients. The reduction in the incidence of postoperative AF was between 23% and 61%. Six studies included 585 patients regarding the efficacy of statins in preventing post-cardioversion AF, but the current evidence does not show a protective effect on the part of statins.

In a retrospective study including 3833 ASCVD patients, Chang et al. [89] aimed to evaluate the efficacy of statins by including patients with AF and a high/very high risk of ASCVD. The results indicated that, in patients with AF, the use of statins was associated with lower rates of achieving LDL-C targets compared to the general population with high and very high cardiovascular risk. Specifically, among high-risk and very high-risk ASCVD patients, the rates of reaching LDL-C management targets were 26.7% and 36.4%, respectively [89].

### 7.2. Bempedoic Acid

Bempedoic acid (BA) is a first-in-class inhibitor of adenosine triphosphate–citrate lyase (ACL) that interferes with an early step in cholesterol synthesis, decreases intrahepatic cholesterol production, upregulates LDL-r expression, and enhances clearance of circulating LDL particles, thus lowering blood LDL-c levels [90]. BA is a prodrug that is converted to its active form by the enzyme of very long-chain acyl-CoA synthetase-1 (ACSVL1), which is expressed primarily in the liver [90].

Cholesterol Lowering via Bempedoic Acid, an ACL-Inhibiting Regimen (CLEAR) Outcomes study was a double-blind, randomized, placebo-controlled trial involving 13,970 statin-intolerant patients who had or were at high risk for cardiovascular disease. The patients were assigned to receive oral BA, 180 mg daily, or a placebo. The primary endpoint was a four-component composite of major cardiovascular events (MACEs), defined as death from cardiovascular causes, nonfatal myocardial infarction, nonfatal stroke, or coronary revascularization. Incidence of a primary endpoint event was significantly lower with BA than with a placebo (11.7% vs. 13.3%; HR, 0.87; *p* = 0.004) [91]. In the CLEAR Outcomes Trial, AF occurred in 3.3% and 3.5% of patients who received BA or a placebo, respectively [91]. AF was balanced compared to the placebo.

Bays et al. [92] described detailed safety information from CLEAR Outcomes, including United States (US) events. AF occurred in 1.2% and 1.4% of patients who received BA or a placebo, with no difference between treatment groups.

### 7.3. Anti-Proprotein Convertase Subtilisin/Kexin Type 9

Proprotein convertase subtilisin/kexin type 9 (PCSK9) is continuously produced within the endoplasmic reticulum of hepatocytes, further modified in the Golgi apparatus, and then released from the cell into the bloodstream. PCSK9 binds to LDL-r, promoting lysosomal degradation. The degradation of the hepatic LDL-r reduces the clearance of plasma LDL, leading to hypercholesterolemia and subsequent atherosclerotic plaque formation [93].

The relationship between PCSK9 and inflammatory markers, including white blood cells (WBC), fibrinogen, and high-sensitivity C-RP, has been evaluated in several experimental studies [94].

In a sepsis mouse model, PCSK9 overexpression increased the systemic release of the inflammatory cytokine IL-6 and exacerbated lung and liver inflammation, whereas PCSK9 deficiency reduced circulating levels of IL-6 and ameliorated organ inflammation [95].

These experimental findings were supported by the results of a human study that included 152 patients in septic shock carrying a PCSK9 loss-of-function allele. Patients with this allele had lower plasma levels of the pro-inflammatory cytokines TNF-α, IL-6, IL-8, and monocyte chemoattractant protein-1 (MCP-1) compared to 21 patients carrying a gain-of-function allele [94].

In arthritis model rats, higher levels of plasma and atrial PCSK9 were observed compared to controls [96]. Arthritis model rats showed a significantly higher AF induction (90%) than control rats (30%) and exhibited a considerably prolonged AF duration. Among this animal cohort, the administration of evolocumab led to a decrease in AF induction and AF duration during electrophysiological study compared to controls [96].

Anti-PCSK9 therapies are fully human monoclonal antibodies (mAbs) that specifically recognize and bind to the PCSK9 protein [93]. When an mAb attaches to circulating PCSK9 molecules, it blocks the active binding site that usually interacts with the LDL receptor. As a result, PCSK9 can no longer bind to and destroy the LDL-r, preventing its recycling back to the cell surface. This mechanism increases LDL receptor expression on the hepatocyte surface, reducing serum LDL levels [93].

#### 7.3.1. Evidence from Clinical Trials

##### Alirocumab

The Evaluation of Cardiovascular Outcomes After an Acute Coronary Syndrome During Treatment With Alirocumab (ODYSSEY OUTCOMES) Trial was a randomized, double-blind, placebo-controlled study that compared the efficacy and safety of alirocumab versus a placebo in 18,924 patients who had an acute coronary syndrome 1 to 12 months earlier and an LDL-c level of at least 70 milligrams (mg) per deciliter, a non−HDL-c level of at least 100 mg per deciliter, or an ApoB level of at least 80 mg per deciliter, and were receiving statin therapy at a high-intensity dose or the maximum tolerated dose. Patients were randomly assigned to receive alirocumab subcutaneously at a dose of 75 mg (9462 patients) or a matching placebo (9462 patients) every 2 weeks. The primary endpoint was a composite of death from coronary heart disease, nonfatal myocardial infarction, fatal or nonfatal ischemic stroke, or unstable angina requiring hospitalization. The median duration of follow-up was 2.8 years. A composite primary endpoint event occurred in 903 patients (9.5%) in the alirocumab group and 1052 patients (11.1%) in the placebo group (HR: 0.85; *p* < 0.001) [8]. Among patients who had previous acute coronary syndrome and who were receiving high-intensity statin therapy, the risk of recurrent ischemic cardiovascular events was lower among those who received alirocumab than among those who received a placebo.

In a post hoc analysis, Lopes et al. [97] aimed to identify factors associated with the development of a first episode of incident AF in patients with recent acute coronary syndrome and to determine whether alirocumab treatment influenced the risk of incident AF. Among 18,262 participants without prior AF at baseline, 499 (2.7%) had incident AF during follow-up. Treatment with alirocumab or a placebo did not significantly influence the cumulative incidence of AF (HR 0.91; 95% CI, 0.77–1.09). Patients with vs. without a history of AF had a higher incidence of MACE (8.8 vs. 3.7 events per 100 patient years), without significant interaction between AF and randomized treatment in terms of the risk of MACE (p_interaction_ = 0.78) [97].

##### Evolocumab

The Further Cardiovascular Outcomes Research with PCSK9 Inhibition in Subjects with Elevated Risk (FOURIER) Trial was a randomized, double-blind, placebo-controlled trial involving 27,564 patients with atherosclerotic cardiovascular disease and LDL-c levels of 70 mg per deciliter or higher who were receiving statin therapy. Patients were randomly assigned to receive evolocumab (140 mg every 2 weeks or 420 mg monthly) or a matching placebo as subcutaneous injections. The primary efficacy endpoint was a composite of cardiovascular death, myocardial infarction, stroke, hospitalization for unstable angina, or coronary revascularization. The key secondary efficacy endpoint was a composite of cardiovascular death, myocardial infarction, or stroke. The median follow-up duration was 2.2 years.

At 48 weeks, the least-squares mean percentage reduction in LDL cholesterol levels with evolocumab, compared with a placebo, was 59% (*p* < 0.001). Evolocumab treatment significantly reduced the risk of the primary endpoint (HR, 0.85; *p* < 0.001) and the key secondary endpoint (HR, 0.80; *p* < 0.001) [9]. Among patients who were receiving high-intensity statin therapy, the risk of cardiovascular events was lower among those who received evolocumab than among those who received a placebo.

Li et al. [98] conducted a summary-level, genome-wide association study, using a single-nucleotide polymorphism linked to lower LDL-C levels as a proxy to simulate the inhibition of PCSK9 and HMG-CoA reductase. Among 173,082 patients with a genetically predicted inhibition of PCSK9, a reduced risk of AF (OR: 0.92; *p* = 0.01) was shown. In contrast, the inhibition of HMG-CoA reductase did not exhibit a statistically significant association with AF risk (OR = 1.11, *p* = 0.05) [98].

In a meta-analysis including 95,635 participants from 26 RCTs, Yang et al. [99] aimed to evaluate whether PCSK9i may attenuate AF progression compared with a placebo or ezetimibe. PCSK9i significantly reduced AF incidence versus a placebo (RR 0.84; *p* = 0.03); in contrast, no significant difference was shown versus the ezetimibe group (RR 0.90; 95%; *p* = 0.85).

Finally, PCSK9i markedly decreased the incidence of cerebrovascular events (RR 0.75; *p* < 0.0001) and new-onset hypertension (RR 0.92; *p* = 0.003), without modifying the risk of cardiovascular death (RR 0.95; *p*= 0.40) and new-onset diabetes mellitus (RR 1.01; *p* = 0.67) [99].

##### Inclisiran

Inclisiran is a small interfering RNA (siRNA) that inhibits PCSK9 synthesis, reducing LDL-c levels and circulating PCSK9.

The ORION-9 [100], ORION-10 [101], and ORION-11 [102] trials were phase 3, double-blind, randomized, placebo-controlled trials that evaluated the efficacy and safety of inclisiran in patients with elevated LDL-c (≥70 mg/dL for patients with ASCVD and ≥100 mg/dL for patients with ASCVD risk equivalent and those with heterozygous familial hypercholesterolemia) despite receiving maximum tolerated statin therapy. Patients were randomized 1:1 to receive 284 mg inclisiran (300 mg inclisiran sodium) or a placebo on days 1 and 90, and every 6 months thereafter; they were followed for 540 days (18 months). The co-primary efficacy endpoints included a percentage change in LDL-c from the baseline to day 510 and a time-adjusted percentage change in LDL-c from the baseline after day 90 and up to day 540. The mean percentage change in LDL-c from the baseline to day 510 in patients with cerebrovascular disease was −45.7% in the inclisiran arm and 9.5% in the placebo arm, resulting in a placebo-corrected difference of –55.2% (95% CI: –64.5 to –45.9; *p* < 0.0001). In a meta-analysis of three RCTs [100,101,102], including 3655 patients (1833 in the inclisiran group and 1827 in the placebo group), no significant association between inclisiran and the incidence of AF (OR, 1.33; *p* = 0.41) was found [100,101,102].

### 7.4. Triglyceride-Lowering Therapies

Omega-3 fatty (O3FA) long-chain polyunsaturated fatty acids (PUFAs), including eicosapentaenoic acid (EPA) and docosahexaenoic acid (DHA), are components of the cell membrane associated with improvement in membrane fluidity [103]. O3FA consumption improves plaque stabilization, reducing the volume of plaque and foam cells [104]. Moreover, O3FA has lipid-effect-reducing apolipoprotein C3, apolipoprotein B, and triglyceride-rich lipoprotein [105]. O3FA also has membrane-stabilizing effects through a reduction in oxidation lipoproteins [106], as well as anti-thrombotic effects via a reduction in cyclooxygenase-2 activity, thromboxane A2 improvement, and endothelial function amelioration through an increase in nitric oxide [107]. Furthermore, PUFA has anti-inflammatory pleiotropic effects, improving prostaglandin synthesis, C-RP, and inflammatory cytokines synthesis of resolvins, protectins, and maresins [108].

In a recent Network Meta-Analysis including 125,763 patients from 14 RCTs, Lombardi et al. showed that showed that high-dose O3FA treatment, defined as 1.8–4 g, was associated with a lower risk of cardiac death (9%), myocardial infarction (16%), sudden cardiac death (12%), unstable angina (16%), and major vascular events (6%) compared to control and low-dose groups. However, high-dose treatment was also linked to an increased risk of bleeding (42%) events and AF (28%) compared to control groups [109].

In contrast, Wilbring et al. [110] showed that 2 g of omega-3 PUFA, initiated 5 days before surgery, reduced the relative risk of postoperative AF by 34.8% in patients with recent myocardial infarction (≤3 months) undergoing isolated coronary artery bypass grafting.

#### 7.4.1. Icosapent Ethyl

Icosapent ethyl (IPE) is a stable ethyl ester of EPA. The mechanisms of action of icosapent ethyl that contribute to reducing cardiovascular events are not fully understood. Icosapent ethyl improves the lipoprotein profile by suppressing cholesterol, fatty acid and TG synthesis enzymes, increasing fatty acid β-oxidation, and reducing microsomal triglyceride transfer protein, resulting in decreased hepatic synthesis and release of TG and VLDL [111]. In addition, icosapent ethyl increases the expression of lipoprotein lipase, resulting in increased removal of TG from circulating VLDL and chylomicrons. In patients with elevated TG levels, IPE reduces TG, VLDL, remnant lipoprotein cholesterol, and levels of inflammatory markers such as C-RP [112].

The Reduction of Cardiovascular Events with Icosapent Ethyl-Intervention Trial (REDUCE-IT) study, a large-scale, double-blind international trial, enrolled 8,179 patients already taking statins with controlled LDL-C levels, elevated TG, and cardiovascular risk factors. These patients were randomly assigned to receive either IPE 4g/day or a mineral oil placebo and were followed for a median duration of 4.9 years [113]. The primary endpoint was a composite of cardiovascular death, nonfatal myocardial infarction, nonfatal stroke, coronary revascularization, or unstable angina. The key secondary endpoint was a composite of cardiovascular death, nonfatal myocardial infarction, or nonfatal stroke.

After 4.9 years of follow-up, IPE treatment resulted in a significant 25% reduction in the primary endpoint and a 26% reduction in the secondary endpoint. Additionally, IPE substantially lowered the risk of individual events, including cardiovascular death (by 20%), stroke (by 28%), myocardial infarction (by 31%), cardiac arrest (by 48%), and sudden cardiac death (by 31%) [113].

However, a more significant risk of hospitalization for AF was noted in patients receiving IPE. Post hoc efficacy and safety analysis by Olshansky et al. [114] examined the relationship between IPE and outcomes in patients with or without prior AF and those with or without in-study AF-related hospitalizations.

The study revealed that hospitalization rates for AF were higher in patients with a history of AF (12.5% in the IPE group versus 6.3% in the placebo group; *p* = 0.007). In contrast, hospitalization rates for AF were similar for those without prior AF (2.2% in IPE versus 1.6% in the placebo group; *p* = 0.09). Serious bleeding events also showed a similar trend, with higher rates in patients with prior AF (7.3% in IPE versus 6.0% in the placebo group; *p* = 0.59), though the differences were not statistically significant for patients without prior AF (2.3% in IPE versus 1.7% in the placebo group; *p* = 0.08). Overall, the investigational product was associated with a higher risk of severe bleeding, regardless of whether the patient had a history of AF (p_interaction_ = 0.61) or developed AF during the study (p_interaction_ = 0.66). Notably, the relative risk reductions with respect to the primary and key secondary composite endpoints with the investigational product versus the placebo were similar between patients with prior AF (9.2% of the study population) and those without prior AF (90.8% of the population), as indicated by the non-significant interaction *p* values (p_interaction_ = 0.37 and p_interaction_ = 0.55, respectively) [114].

#### 7.4.2. Future Directions

Looking ahead, future research should aim to deepen our understanding of the mechanisms through which lipid-lowering therapies influence AF pathophysiology, providing valuable insights into their potential roles in AF prevention and management. To fully realize this objective, collaborative, large-scale clinical trials and long-term follow-up studies should be performed to assess the arrhythmic outcomes of both established and novel lipid-lowering agents in different clinical settings. Moreover, emerging lipid-modifying strategies, including gene therapy and CRISPR-based gene editing [115], present exciting prospects for the precise modulation of lipid profiles and could offer new opportunities to mitigate AF risk. Exploring the genetic and molecular pathways linking lipid metabolism and AF allows us to apply a patient-centered approach and define personalized treatment strategies for optimizing care for patients with coexisting dyslipidemia and AF.

## 8. Conclusions

Systemic inflammation, oxidative stress, and autonomic dysfunction represent the pathophysiological link between dyslipidemia and atrial fibrillation. The presence of AF does not appear to influence the clinical efficacy of lipid-lowering therapies in patients with ASCVD. Furthermore, lipid-lowering therapies seem to be associated with a reduced incidence and recurrence of AF.
